# Ectopic *hbox12* Expression Evoked by Histone Deacetylase Inhibition Disrupts Axial Specification of the Sea Urchin Embryo

**DOI:** 10.1371/journal.pone.0143860

**Published:** 2015-11-30

**Authors:** Vincenzo Cavalieri, Giovanni Spinelli

**Affiliations:** 1 Department of Biological, Chemical and Pharmaceutical Sciences and Technologies (STEBICEF), University of Palermo, Italy; 2 Mediterranean Center for Human Health Advanced Biotechnologies (CHAB), University of Palermo, Italy; Laboratoire de Biologie du Développement de Villefranche-sur-Mer, FRANCE

## Abstract

Dorsal/ventral patterning of the sea urchin embryo depends upon the establishment of a Nodal-expressing ventral organizer. Recently, we showed that spatial positioning of this organizer relies on the dorsal-specific transcription of the Hbox12 repressor. Building on these findings, we determined the influence of the epigenetic milieu on the expression of *hbox12* and *nodal* genes. We find that Trichostatin-A, a potent and selective histone-deacetylases inhibitor, induces histone hyperacetylation in *hbox12* chromatin, evoking broad ectopic expression of the gene. Transcription of *nodal* concomitantly drops, prejudicing dorsal/ventral polarity of the resulting larvae. Remarkably, impairing *hbox12* function, either in a spatially-restricted sector or in the whole embryo, specifically rescues *nodal* transcription in Trichostatin-A-treated larvae. Beyond strengthen the notion that *nodal* expression is not allowed in the presence of functional Hbox12 in the same cells, these results highlight a critical role of histone deacetylases in regulating the spatial expression of *hbox12*.

## Introduction

Information for the ontogeny of a developing organism is interpreted through the hierarchical expression of cohorts of transcription factors and their specific binding to proper genomic target sites. Over developmental time, the interconnections among these molecular cascades assume the architecture of a network and impose transient spatial and temporal regulatory states, which eventually lead to the regional segregation into distinct embryonic territories [1–3].

Pioneer studies in the sea urchin embryo have deciphered the complex Gene Regulatory Network (GRN) that governs patterning of the endomesodermal territories [2]. More recently, the identification of *nodal* and the contribution of large-scale studies allowed dissecting the GRN that accounts for patterning of the sea urchin embryo along the dorsal/ventral (DV) axis [4–12].

DV polarity relies on a combination of inherited maternal information and inductive interactions among early blastomeres, allowing the institution of a ventrally-localized organizer expressing Nodal, a pivotal regulator of the DV GRN [13].

Direct and indirect targets of Nodal signalling include almost thirty genes encoding transcription factors and signal transduction molecules [11,12,14]. Among these, *nodal* itself is subjected to a positive feedback loop related to the short-range Nodal signal transduction system [9,10]. Hierarchically downstream, the genes coding for both BMP2/4 and Lefty are spatially co-expressed with respect to Nodal, and together with the latter constitute the core of the DV network. BMP2/4 ligand diffuses toward the opposite side of the embryo, where it acts as a relay to specify the dorsal ectoderm [4,5]. BMP2/4 signaling activity is confined in this territory due to the inhibition of ligand reception by Chordin within the ventral ectoderm [4,5,15–17]. Lefty is instead a Nodal antagonist that limits Nodal signaling to the ventral ectoderm. As mentioned, Lefty and Nodal are produced by the same cells, but the former is thought to diffuse more rapidly, thus acting as a long-range feedback inhibitor of Nodal [5,18,19].

Foregoing Lefty production, a significant role in shaping the spatial domain of *nodal* expression is played by the Hbox12 transcription repressor, which is expressed by cells that are fated to become dorsal ectoderm, preceding the onset of *nodal* expression. We have recently shown that Hbox12 functions to prevent the ectopic activation of *nodal* transcription by means of dorsal-specific inhibition of the p38-MAPK activity, which is known to be required for *nodal* expression [20]. In this scenario, Hbox12 represents the earliest known zygotic regulator expressed by non-organizer cells and embedded in the GRN that governs polarization along the DV axis of the sea urchin embryo.

To date, only few other developmental GRNs have been described at such a satisfactory level [21–26]. In all models, gene expression is essentially consequential to the integration among transcription factors specifically bound to their cognate *cis*-regulatory elements. On the other hand, packaging of DNA into chromatin imposes additional layers of gene regulation. For instance, the expression state of a given gene largely correlates with the histone post-translational modifications imposed on nucleosomes wrapping the *cis*-regulatory regions of the gene. Among these modifications, acetylation primarily associates with the expression of target genes, and it is fine-tuned through histone acetyltransferases (HATs) and deacetylases (HDACs) [27]. The latter enzymes are generally thought to be transcriptional co-repressors required for essential biological processes, including embryo development [28–34]. However, few early reports explored the relationship between histone acetylation status and transcriptional competence in the sea urchin embryo [35–37].

In the present study, we assessed whether perturbation of the epigenetic milieu by inhibition of HDACs activity affects the expression of *hbox12* and *nodal* genes. We find that Trichostatin-A (TSA), a potent and selective inhibitor of HDACs [38], induces histone hyper-acetylation in *hbox12* chromatin, provokes ectopic expression of the gene across the embryo, and, in turn, hinders *nodal* expression. Finally, impairing *hbox12* function specifically rescues *nodal* expression in TSA-treated embryos, emphasizing the notion that *nodal* expression is not allowed in the presence of functional Hbox12 in the same cells.

## Results

### Treatment with HDAC inhibitors triggers ectopic hbox12 expression

**Fig 1:**
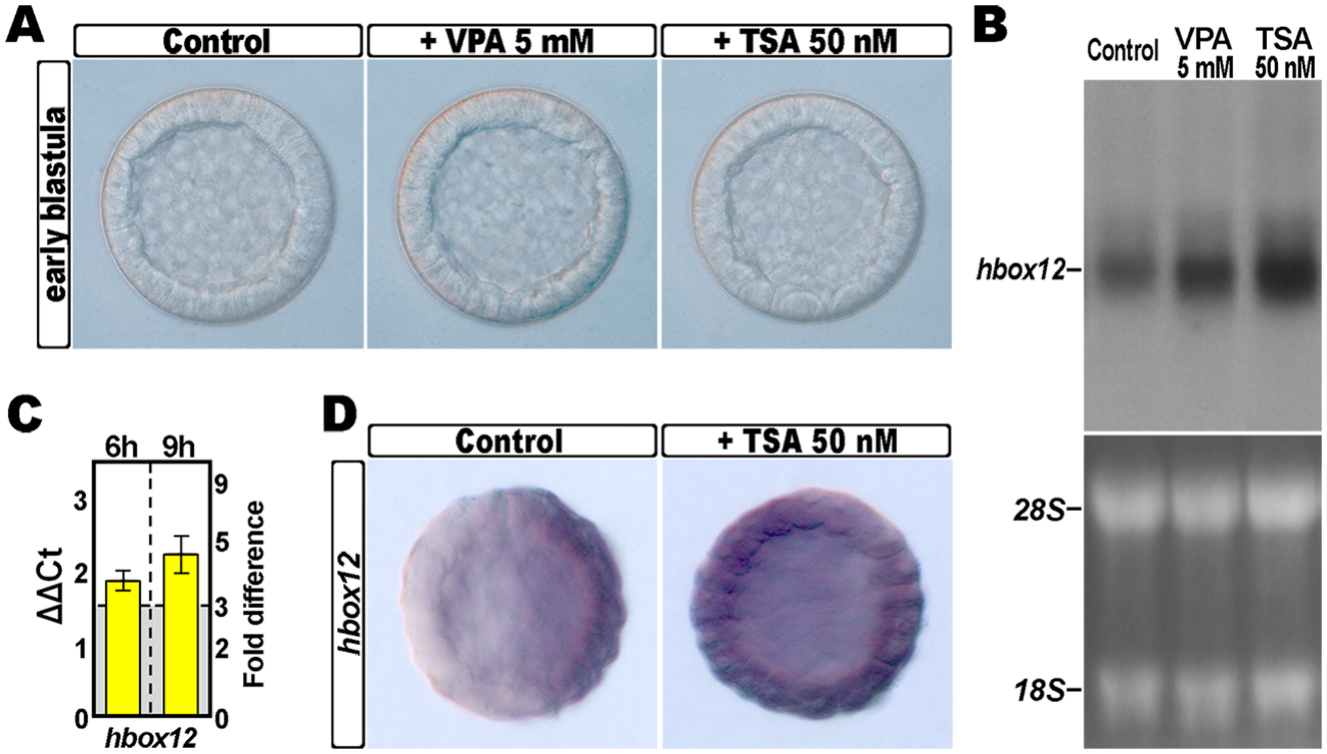
Effect of HDAC inhibition on the expression of the *hbox12* gene. (A) *P*. *lividus* embryos cultured in the absence or in the presence of either TSA or VPA at the indicated dosages, and observed at the early blastula stage. (B) Northern blot analysis of total RNA isolated from embryos at the early blastula stage treated or not treated with TSA or VPA, and probed with an antisense ^32^P labelled RNA against the *hbox12* transcript. The lower panel shows the loading control ribosomal RNAs in the ethidium bromide stained agarose gel. (C) qPCR measurements of *hbox12* transcript abundance in blastulae treated with 50 nM TSA. Data are shown as normalized ΔCt (ΔΔCt, left ordinate), and as the corresponding fold difference in transcript abundance (right ordinate), with respect to control unperturbed embryos at the same stages of development. The gray region represents ΔΔCt values corresponding to less than 3-fold difference. Error bars are standard errors for the qPCR replicates. Oligonucleotide primer pairs used for qPCR reactions and amplicon lengths are indicated in the S1 Table. (D) Spatial distribution of the *hbox12* transcripts in control and TSA-treated embryos at the early blastula stage, revealed by chromogenic WMISH.

To investigate whether histone acetylation is involved in the activation of *hbox12* gene expression during sea urchin development, *Paracentrotus lividus* embryos were cultured in the presence of the HDAC inhibitors TSA or valproic acid (VPA). Treatment started from fertilization at concentrations of 50 nM and 5 mM, respectively. At these dosages, that are commensurate with effective doses determined in studies in mammalian systems [39,40], the rate of cell division was not altered, and embryos cleaved synchronously with respect to untreated controls (Fig 1A).

Under these experimental conditions, *hbox12* transcript level increased when compared to controls, as determined by Northern blot assay (Fig 1B). Such an increase in *hbox12* mRNA abundance was much higher with TSA than VPA, which is in line with the reported stronger inhibition of HDACs by TSA [41]. For this reason, the effect of TSA on *hbox12* expression was further investigated. qRT-PCR measurements revealed that the overall *hbox12* mRNA amount in embryos exposed to TSA was at least three fold higher than that of control embryos throughout cleavage (Fig 1C).

As HDAC inhibition results in hyper-acetylation of hystones [42,43], which in turn is generally associated to transcriptionally active chromatin [44], we reasoned that the observed upregulation of *hbox12* expression could reflect the additional transcription of the gene in ectopic territories. Whole mount in situ hybridization (WMISH) indeed confirmed that *hbox12* was ectopically expressed across the TSA-treated early blastulae (Fig 1D). By contrast, and as expected, *hbox12* transcripts were detected exclusively in prospective dorsal ectoderm cells of control unperturbed embryos observed at the same stage (Fig 1D) [20].

### TSA treatment increases acetylation of histone H3 associated with the hbox12 promoter

As mentioned, it is well documented that HDAC inhibitors induce a global increase of histone acetylation. Particularly, acetylation of histone H3K9 has been proposed as a signature of active transcription, as it is found principally enriched in the 5’ *cis*-regulatory region of expressed genes, where it creates an accessible chromatin domain [45,46].

Therefore, we assessed whether the TSA-induced overexpression of *hbox12* correlated with an increased level of acetylated H3K9 (H3K9ac) at the promoter region of the gene.

**Fig 2:**
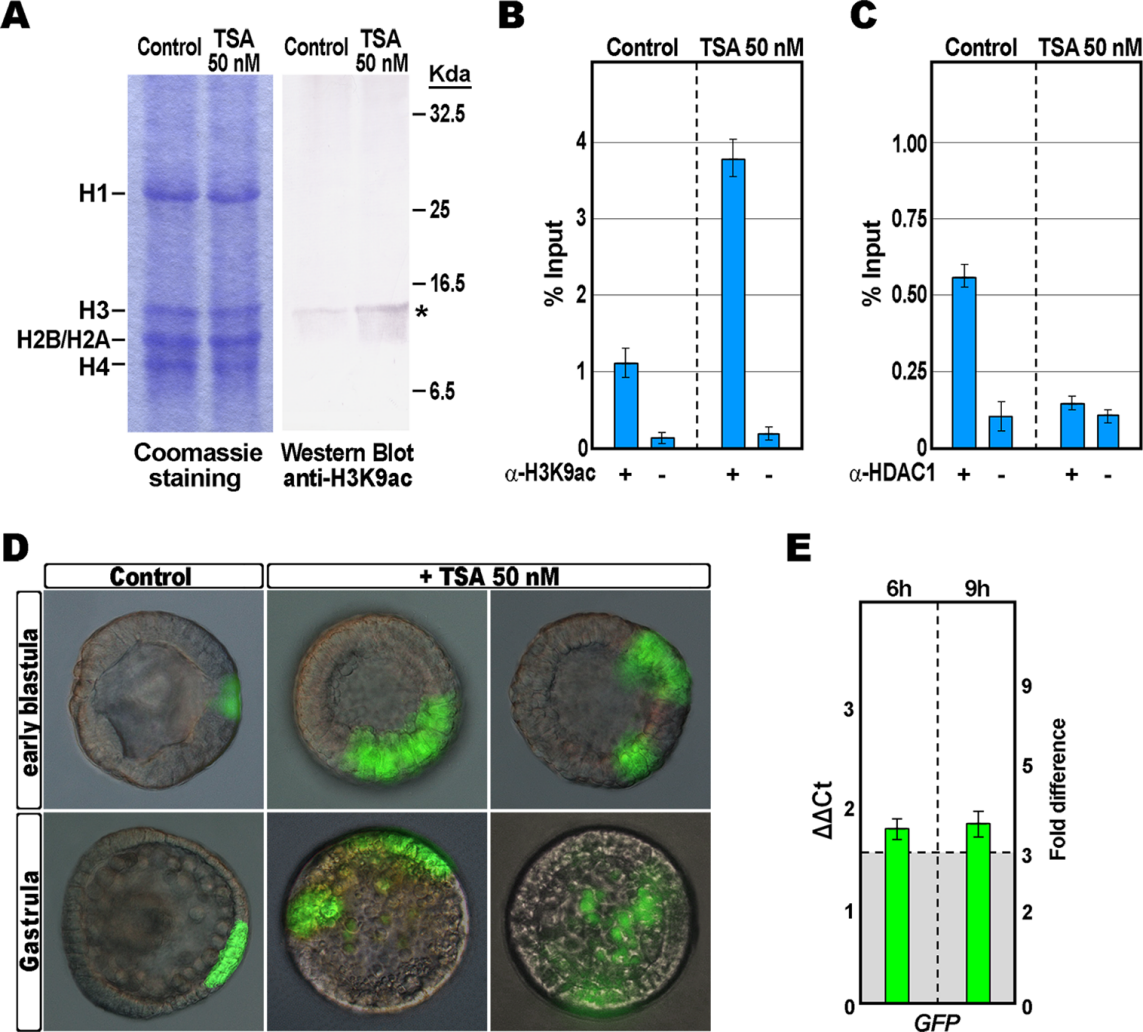
Impact of TSA on acetylation of H3K9 and *hbox12* promoter activity. (A) Western blot analysis using the anti-H3K9ac antibody. Nuclear extracts from control and 50 nM TSA-treated blastula stage embryos were fractioned by SDS-PAGE (on the left is shown the gel staining), blotted on PVDF membrane and incubated with anti-H3K9ac antibody. (B and C) ChIP-qPCR analysis of the *hbox12* promoter occupancy by H3K9ac and HDAC-1. ChIP assays were performed on chromatin extracted from control and TSA-treated embryos at the mesenchyme blastula stage and precipitated with commercial antiserum against H3K9ac or HDAC-1, or incubated without adding antibodies, followed by qPCR amplification of an *hbox12* promoter fragment. Data are normalized according to the percent of input method. Bars are as in Fig 1C. (D) Zygotes were injected with the *phbox12*-GFP transgene, the resulting embryos were raised in the presence of TSA 50 nM, and observed at the indicated stages. Images for each embryo are shown under DIC optic superimposed on epifluorescence field. (E) qPCR measurements of *gfp* transcript abundance in blastulae treated with 50 nM TSA. Data are normalized and indicated as in Fig 1C.

First, by western blot analysis carried out using a specific antibody that recognizes H3K9ac, we ascertained that mesenchyme blastulae treated with 50 nM TSA accumulated a global increase in the acetylated H3 level compared to that of control embryos at the same stage (Fig 2A). Next, quantitative ChIP assays with the anti-acetyl-H3K9 antibody were performed on chromatin purified from mesenchyme blastulae treated or untreated with TSA. As expected, no or faint amplification was detected for the chromatin samples incubated without antibody, used as a negative control (Fig 2B). By contrast, the amplification of a specific DNA segment from the *hbox12* promoter clearly demonstrated that it was heavily enriched in acetylated histone H3 in TSA-treated embryos (Fig 2B). Thus, TSA likely exerted a direct effect on histone acetylation at the *hbox12* promoter.

Since in conventional deacetylation assays TSA showed the highest potency towards HDAC-1 [47], we appraised the recruitment of HDAC-1 on the *hbox12* promoter by performing a subsequent ChIP assay on sister samples of chromatin, using a commercial antibody. We reported previously that this antibody reacts in nuclear extracts with a single protein band of the expected size for the sea urchin HDAC-1, and that it specifically recognizes the *P*. *lividus* HDAC-1 expressed in *E*. *coli* from the cloned gene [35]. The occupancy of HDAC-1 was unequivocally mapped on the *hbox12* promoter in chromatin derived from control unperturbed embryos at the mesenchyme blastula stage, coherent with the transcriptional shutoff of the gene (Fig 2C) [20,48]. Strikingly, HDAC-1 recruitment was instead prevented by TSA treatment (Fig 2C).

To better investigate the relationship between HDAC-1 inhibition and *hbox12* gain of transcription, we examined whether TSA could activate the *hbox12* promoter in gene transfer assays. We used a *phbox12*-GFP transgene containing 1.45 kb of the promoter sequence and the ATG start codon of *hbox12* fused in frame with the GFP coding sequence [20,49]. Such a transgene was injected into developing zygotes that were then reared in the absence or in the presence of TSA 50 nM.

In agreement with previous observations indicating that *phbox12*-GFP accurately recapitulates the early dorsal-specific expression of *hbox12* [20,49], transgene expression was detected during early embryogenesis, and at the gastrula stage green fluorescence was specifically restricted to the dorsal ectoderm of an average of 62% (n>400) of unperturbed injected embryos (Fig 2D).

Following TSA treatment, *phbox12*-GFP expression started at a similar time with respect to control embryos. However, GFP fluorescence was detected in markedly larger and/or double patches throughout development of TSA-treated embryos (Fig 2D). Development of these embryos was rather normal until gastrulation (Fig 2D). By this time, control embryos displayed a clear DV polarity as shown by the thickening of the ventral side and the symmetric ventral-lateral arrangement of the two primary mesenchyme cell (PMC) clusters (Fig 2D). In striking contrast, more than 70% (n>500) of embryos exposed to TSA remained almost spherical, did not gastrulate, their mesenchyme cells were irregularly dispersed into the blastocoel, and skeletal elements were not mineralized (Figs 2D and 4D). Similar teratogenic effects on embryo development have been reported for other HDAC inhibitors [50–52].

Intriguingly, the observed phenotype was somewhat similar to that imposed by the ubiquitous expression of the synthetic *hbox12* mRNA injected into developing zygotes [20]. Accordingly, more than 70% (n>500) of the *phbox12*-GFP-injected and TSA-treated embryos expressed the transgene ectopically (Fig 2D). qRT-PCR measurements of the *gfp* mRNA abundance in *phbox12*-GFP injected embryos at the early blastula stage predictably revealed a notable increase specifically associated to TSA exposure (Fig 2E).

### TSA-induced overexpression of hbox12 downregulates nodal transcription

The results showed in the previous sections collectively indicate that TSA treatment evokes massive and ectopic expression of *hbox12* across the embryo. Based on our previous findings highlighting the negative functional connection between *hbox12* and *nodal* genes [20], we syllogistically inferred that exposure of developing embryos to TSA could result in weakening of *nodal* expression.

**Fig 3:**
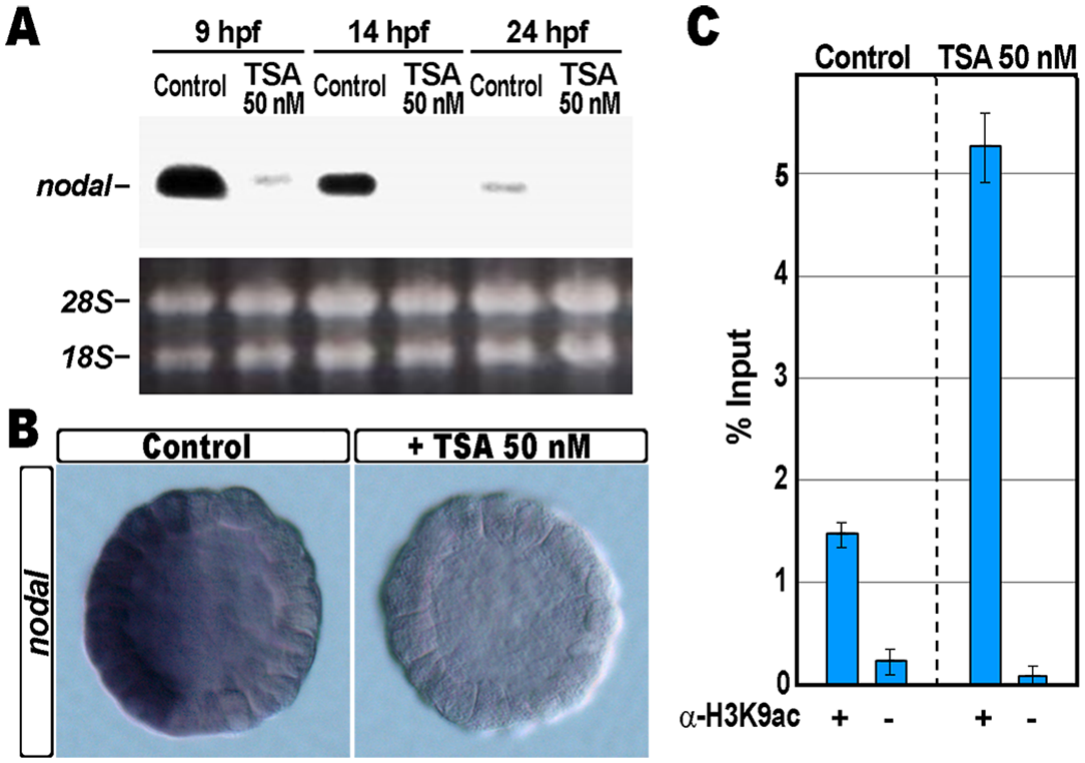
TSA treatment and effect on *nodal* gene expression. (A) Northern blot analysis of total RNA isolated from embryos at the indicated stages exposed or not to TSA 50 nM, and probed with an antisense ^32^P labelled pRNA against the *nodal* transcript. The lower panel shows the loading control ribosomal RNAs in the ethidium bromide stained agarose gel. (B) Control and TSA-treated embryos were fixed at the early blastula stage, and analysed by WMISH with a DIG-labelled *nodal* probe. (C) ChIP-qPCR analysis of the *nodal* promoter occupancy by H3K9ac. As a negative control, chromatin samples were incubated without antibodies, and negligible amplification was obtained from the corresponding purified DNA. Data are normalized according to the percent of input method, and shown as in Fig 2B and 2C.

In accordance with this prediction, residual transcription of *nodal* occurred in TSA-treated blastulae, and it was completely abrogated in embryos at later stages of development, as indicated by Northern blot assay (Fig 3A). Likewise, while *nodal* transcripts were accumulated exclusively by prospective ventral ectoderm cells of all control embryos at the blastula stage (Fig 3B), by WMISH we did not observe detectable expression of *nodal* in the vast majority of the TSA-treated embryos at the same stage (86%, n = 182; Fig 3B).

Despite the above mentioned drop of transcription, ChIP assays pointed out that significant H3K9 hyper-acetylation was specifically incorporated on the *nodal* gene promoter following exposure of the embryos to TSA (Fig 3C). This is not surprising, because a high histone acetylation level only indicates better accessibility of the chromatin locus, not necessarily reflecting a positive correlation with gene transcription.

### Impairing hbox12 function restores nodal expression in TSA-treated embryos

**Fig 4:**
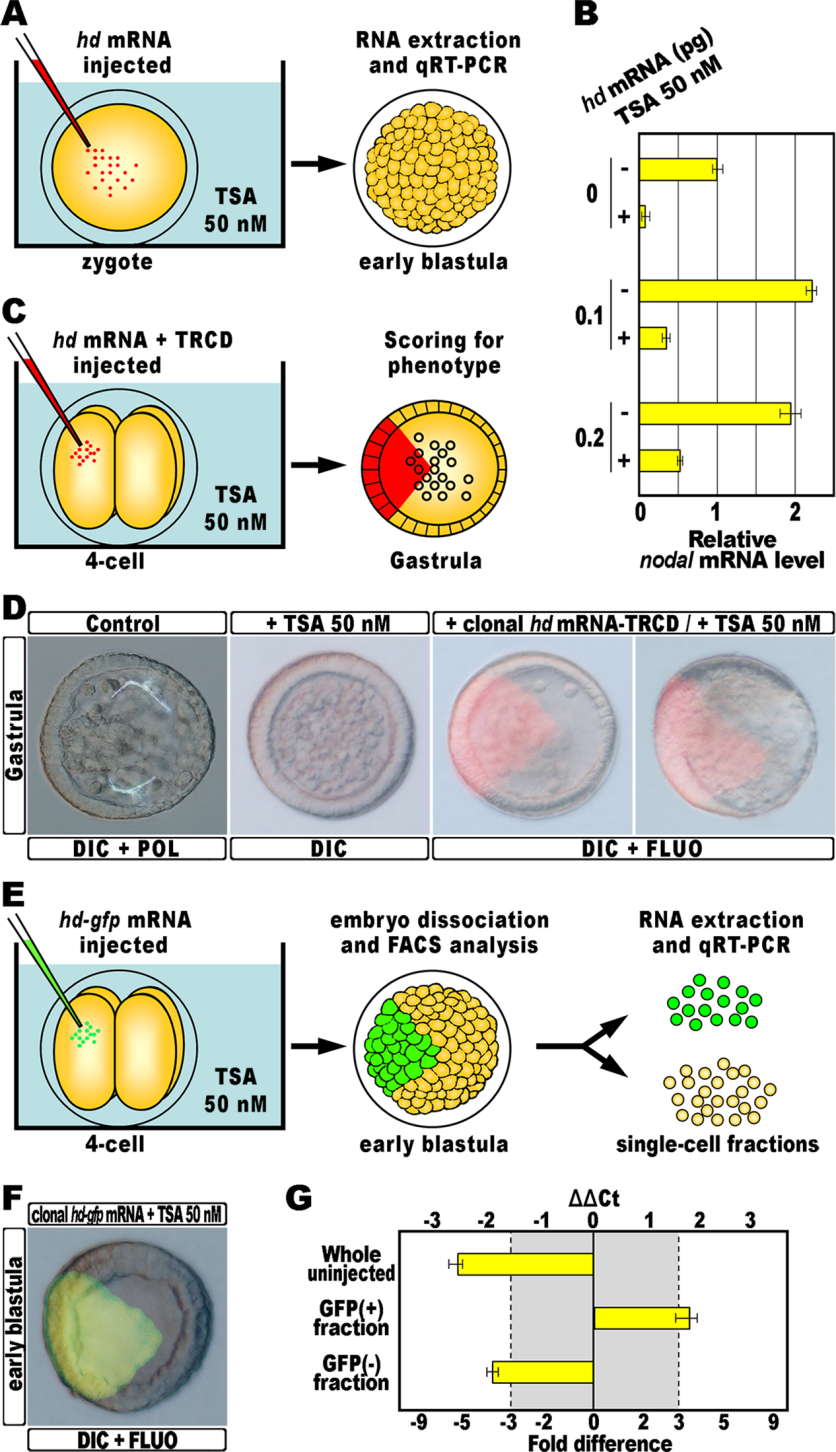
Rescue of *nodal* transcription by HD expression into TSA-treated embryos. (A) 0.1–0.2 pg of the *hd* mRNA, or a control out-of-frame *strim1* transcript [53], were injected into zygotes exposed to TSA and total RNA isolated from the resulting embryos at the early blastula stage. (B) qPCR measurements of relative *nodal* transcript abundance in embryos exposed to TSA and injected with increasing amounts of the *hd* mRNA, compared to the *nodal* mRNA level of control unperturbed embryos. Data are normalized and indicated as in Fig 1C. (C) At the 4-cell stage, one blastomere of TSA-treated embryos was injected with the *hd* mRNA together with the TRCD red fluorescent tracer, and the phenotype of the resulting embryos was examined at the gastrula stage. (D) Representative examples of control gastrulae, TSA-treated larvae, and rescued embryos at the same stage injected with the *hd* mRNA, respectively ordered from left to right. Note that in both the rescued embryos, the progeny of the blastomere that received *hd* was embedded into the ventral side. (E) At the 4-cell stage, one blastomere of TSA-treated embryos was injected with the *hd-gfp* mRNA, and the resulting embryos at the early blastula stage were disaggregated into individual cells that were eventually segregated into two populations, based on GFP fluorescence, by way of FACS. (F) Representative example of embryos expressing HD-GFP clonally, observed at the early blastula stage, just before dissociation. (G) Changes in gene expression level of *nodal* assessed by qPCR in whole embryos exposed to TSA, and in subpopulations of cells sorted from rescued TSA-treated embryos clonally expressing HD-GFP. Data are normalized and indicated as in Fig 1C.

To endorse the specificity of the functional relationship between *hbox12* and *nodal*, we performed a rescue assay in which the overexpression of the isolated homeodomain of Hbox12, referred to as HD, was inflicted to developing embryos exposed to TSA. The experimental assay is depicted in Fig 4A. The *hd* mRNA, or the control out-of-frame *strim1* RNA [53], was microinjected into zygotes that were then cultured in the presence of TSA 50 nM until the early blastula stage, and eventually processed by qRT-PCR. Since we have previously shown that HD efficiently competes with the endogenous Hbox12 for binding to target DNA sequences [20], we reasoned that a molar excess of HD could counteract the TSA-induced overexpression of *hbox12*, restoring *nodal* transcription to some extent. In full agreement, the prevalence of the *nodal* mRNA specifically raised with the injection of increasing amounts of the *hd* transcript (Fig 4B).

Based on this result, we considered that the localized expression of HD should concomitantly allow restricted expression of *nodal*. To confirm this prediction, the *hd* mRNA was injected into a single randomly chosen blastomere of 4-cell stage embryos exposed to TSA (Fig 4C). To follow the fate of the injected cells, the *hd* mRNA was delivered together with the Texas Red conjugated dextran (TRCD) tracer. Sister batches of zygotes were cultured in the absence or presence of 50 nM TSA, and observed at 30 hours post-fertilization. At this stage, unperturbed embryos displayed a clear DV polarity as shown by the thickening of the ventral ectoderm, the bending of the archenteron towards the oral ectoderm, and the symmetric ventral-lateral arrangement of the two primary mesenchyme cell clusters, each embedding a triradiate spicule (Fig 4D). Once again, TSA-treated embryos observed at the same stage were instead quite rounded and devoid of both archenteron and skeletal elements (Fig 4D). Most notably, these embryos appeared to be constituted by a uniformly thickened epithelium (Fig 4D). In striking contrast, clonal expression of HD renewed the unequal ectodermal thickness in most of the resulting TSA-treated embryos (80%, n = 218; Fig 4D), suggesting that polarization of the ectoderm probably occurred to some extent. Remarkably, inspection of these larvae under fluorescence illumination clearly revealed that the progeny of the blastomere injected with the *hd* mRNA was invariably found on the sector with ventral morphological features (Fig 4D).

To ascertain that the HD-expressing cells were the only source of *nodal* in TSA-treated larvae, a single blastomere of 4-cell stage embryos exposed to TSA was injected with a synthetic mRNA coding for a HD-GFP fusion protein, that it has been demonstrated to be functionally equivalent to HD alone [20]. The resulting embryos were cultured in the presence of 50 nM TSA until the early blastula stage, disaggregated to individual cells, and immediately sorted based on the GFP fluorescence by flow cytometry (Fig 4E and 4F).

Then, the mRNA abundance of *nodal* was examined by qRT-PCR and, as expected, upregulation was specifically detected in samples derived from the fluorescent fraction (Fig 4G). Importantly, the reciprocal population consisting of non-fluorescent cells derived from the same embryos had significantly downregulated *nodal* expression to a similar extent of the whole uninjected TSA-treated embryos (Fig 4G).

We conclude that the localized knock-down of Hbox12 was able to restore the asymmetrical transcription of *nodal* in TSA-treated embryos.

## Discussion

Despite the intellectual explanatory power of GRNs, the comprehension of the molecular mechanisms that govern embryonic development is far away from complete. In recent years, a plethora of studies have emphasized that the epigenetic framework by which genes are harnessed could also control the time and place of the transcriptional event.

In this study we focused on how inhibition of histone deacetylation impinges on the expression of two genes, *hbox12* and *nodal*, located at the top of the GRN governing the formation of the DV axis of the sea urchin embryo. In particular, we found that inhibition of HDAC activity by means of exposure of developing embryos to TSA or VPA increases the amount of acetyl-H3K9 associated with the *hbox12* promoter (Fig 2B). Previous reports highlighted that class I and II, but not III, HDAC enzymes are susceptible to inhibition by both compounds, although TSA has the highest potency towards HDAC-1 [47,54,55].

Our results indicate that HDAC-1, the prototypical class I enzyme, is normally recruited at the *hbox12* locus, and that TSA treatment nullifies this connection (Fig 2C). In agreement with this observation, CEM-CCRF cells consistently reduced HDAC-1 binding on the *MDR1* gene promoter following exposure to TSA [56]. In another closely related case, TSA treatment of MCF-7 cells elicited the clearance of the repressive HDAC-1/HDAC-2/mSin3A complex from the *LHR* promoter, resulting in the local accumulation of hyper-acetylated histones [57]. The comparable binding of HAT enzymes to the *LHR* promoter, regardless the presence of TSA, revealed that the release of the HDAC-containing complex is critical for skewing the acetylation balance towards a global hyper-acetylation state affecting chromatin assembly [43,57]. Similarly, this would imply that, in the absence of functional HDAC-1, HAT activities probably dominate also on the *hbox12* promoter.

Previous reports have shown that the effects of HDAC inhibitors on gene expression are not global but they rather impact against a fraction of selected genes within the genome (10% to 40%), with a comparable number of responsive genes being repressed or derepressed [58–61]. Gene transfer assays support the contention that the increase in acetylated nucleosomes stimulates *hbox12* promoter activity, in turn eliciting ectopic expression of the gene across the embryo (Figs 1 and 2). Thus, *hbox12* belongs to the group of genes that are up-regulated by HDAC inhibitors. We speculate that this outcome could occur because an unidentified regulator localized on the ventral side of the embryo requires HDAC-1 to function as a spatial repressor of *hbox12* expression. This hypothesis is supported by several studies describing that many transcription factors that act as repressors indeed recruit HDACs as co-regulatory factors in order to locally prevent activation of a particular gene [62]. Alternatively, the HDAC-1 activity could be itself specifically confined to the embryonic territories that normally do not express *hbox12*. Of outmost relevance, the sea urchin HDAC-1 transcripts are present throughout the sea urchin embryogenesis, being spatially restricted in the endoderm and ventral ectoderm territories [63]. In a more complex scenario, HDAC-1 could also contribute to the territorial repression of *hbox12* through specific deacetylation of a hypothetic negative regulator acting upstream of *hbox12*. In this regard, a number of non-histone proteins, especially nuclear proteins, have been also shown to be regulated through their acetylation status by HDAC activity [64,65].

Whatever is the mechanism, opening of the *hbox12* silenced chromatin in non-dorsal cells affects *nodal* expression, recapitulating what it has been achieved following the injection of the exogenous *hbox12* mRNA (Fig 1C) [20]. Strikingly, despite the TSA-induced hyper-acetylation of H3K9 in nucleosomes wrapping the promoter sequence of *nodal* (Fig 3C), we observed a negative outcome on *nodal* transcription. A reasonable interpretation of this result is that TSA, by means of the augmented histone acetylation level, increased chromatin accessibility of the *cis*-regulatory elements at *nodal* promoter, making it more vulnerable to the overbearing repression triggered by the ectopically expressed Hbox12.

While transcription of *nodal* was drastically abrogated by TSA treatment, the phenotype of the resulting embryos did not exactly coincide with that of *nodal* morphant embryos at gastrula stage [5], with a major difference consisting in the lack of the archenteron in TSA-treated embryos (Figs 2D and 4D). A similar inhibition of gastrulation has been reported during development of starfish [51] and *Xenopus* [50] embryos exposed to TSA at nanomolar concentrations, and it likely reflects a pleiotropic repercussion inflicted by TSA. Beyond the absence of archenteron, however, the ectoderm of TSA-treated embryos did not partition into morphologically distinguishable domains and did not exhibit any sign of DV polarization (Figs 2D and 4D), consistent with the lack of *nodal* expression.

Remarkably, the specificity of the functional link between *hbox12* and *nodal* genes was even further strengthen by the rescue assays of *nodal* expression based on microinjection of the isolated homeodomain of Hbox12, viz HD, into developing embryos exposed to TSA. In the former assay, the ubiquitous expression of increasing amounts of HD specifically counteracted the TSA-induced gain of *hbox12* function, leading to a dose-dependent increase of *nodal* expression (Fig 1A and 1B). In a subsequent rescue assay, the localized expression of HD partially rescued DV polarization of TSA-treated embryos (Fig 4C and 4D). In fact, a key finding emerged by the observation of the rescued embryos at the blastula stage is that the clone of epithelial cells expressing HD invariably assumed morphological features characteristic of ventral ectoderm (Fig 4D), strongly suggesting that *nodal* expression specifically occurred in those cells. In support of this notion, it has been shown that the *nodal* expressing territory belongs exclusively to the presumptive ventral ectoderm of the undisturbed early embryo, and that *nodal* expression promotes ventral fate [5]. In a third rescue assay, by FACS analysis followed by qRT-PCR, we definitively demonstrated that the sector of the rescued embryos expressing a GFP-tagged HD specifically accumulated *nodal* transcripts regardless the overexpression of *hbox12* provoked by TSA (Fig 4E–4G).

Altogether, the data presented here validate our previous model implying that *hbox12* precludes *nodal* transcription within the prospective dorsal ectoderm, thereby acting as a key upstream gene in patterning the DV axis of the sea urchin embryo. Our findings also lead to a better understanding of the molecular basis that control DV polarization and open avenues to pursue future research on the epigenetic mechanisms intersecting gene regulatory networks.

## Material and Methods

### RNA extraction, Northern blot and Whole Mount In Situ Hybridization

Total RNA from embryos at the early blastula stage was extracted by using the RNeasy Midi kit (Qiagen), according to the manufacturer’s instructions. Samples of total RNA (20 μg per lane) were fractioned on 1.5% agarose gel containing 0.66 M formaldehyde, transferred onto a Hybond-N^+^ nylon membrane (Amersham), and cross-linked to the membrane using an UVC 500 crosslinker (Amersham Biosciences). The membrane was hybridized with an antisense ^32^P-labelled *hbox12* RNA, in ULTRAhyb hybridization buffer (Ambion). Stringent washes were performed in 0.1X SSC at 65°C and the membrane was finally subjected to autoradiography using an X-Omat AR film (Kodak).

Chromogenic whole mount in situ hybridization procedure was performed as described [20,53], with Digoxigenin-labeled antisense RNA probes and staged embryos.

### SDS-page and Western blot

Embryos at the mesenchyme blastula stage were harvested by spun at 800 g for 5 min, washed twice in Ca^++^ and Mg^++^ free Millipore filtered sea water, and incubated in cell lysis buffer (10 mM HEPES pH 8.0, 85 mM KCl, 0.5% NP-40, 1 mM PMSF) for 10 min on ice. Pelleted nuclei were then resuspended in nuclear lysis buffer (2M NaCl, 10 mM Tris-HCl pH 7.5, 1 mM PMSF), incubated for 10 min on ice, diluted by adding 1 volume of 1x TE and eventually sonicated using a Bandelin Sonopuls ultrasonic homogenizer. After removing the insoluble materials, the supernatants were analyzed on 24% SDS-polyacrylamide gel electrophoresis. The gel was then stained with Coomassie brilliant blue, destained in methanol/acetic acid, and photographed.

For western blotting, the electrophoresed proteins were transferred onto an Immobilon-P PVDF membrane (Millipore). The membrane was probed with a 1:4000 dilution of anti-acetyl-H3K9 (Upstate cell signaling solutions; cat# 07–352) in 5% nonfat milk, PBS, 0.1% Tween-20, followed by incubation with an alkaline phosphatase conjugated anti-rabbit secondary antibody (Promega) and substrate solution (Roche).

### Reverse transcription and quantitative PCR

Reverse-transcription and qPCR analysis was performed as described [20,66,67]. Briefly, total RNA from batches of embryos grown at the desired stage was extracted by using the Power SYBR Green Cells-to-CT kit (Ambion) and reverse transcribed following the manufacturer’s recommendations. The resulting cDNA samples were further diluted and the equivalent amount corresponding to one embryo was used as template for qPCR analysis, using the oligonucleotide primers indicated in the S1 Table.

qPCR experiments were performed from two distinct batches and all reactions were run in triplicate on a 7300 Real-Time PCR system (Applied Biosystems) using SYBR Green detection chemistry. ROX was used as a measure of background fluorescence and, at the end of the amplification reactions, a ‘melting-curve analysis’ was run to confirm the homogeneity of all amplicons. Calculations from qPCR raw data were performed by the RQ Study software version 1.2.3 (Applied Biosystems), using the comparative Ct method. Primer efficiencies (i.e., the amplification factors for each cycle) were found to exceed 1.9. In every experiment, a no-template control was included for each primers set. A *cytochrome oxidase* or the *mbf1* mRNA, which are known to be expressed at a constant level during development [49,66], were used to normalize all data, in order to account for fluctuations among different preparations.

### Chromatin immunoprecipitation

ChIP experiments were performed essentially as described previously [67]. Briefly, *P*. *lividus* embryos treated or untreated with TSA 50 nM were harvested at the mesenchyme blastula stage, fixed by adding 1% formaldehyde directly to the sea water, and incubated for 10 min at room temperature. Cross-linked embryos were washed three times with ice-cold PBS, collected by centrifugation and incubated in cell lysis buffer containing protease inhibitor (10 mM HEPES pH 8.0, 85 mM KCl, 0.5% NP-40, 1 μg/ml leupeptin, 1 μg/ml aprotinin, 1 mM PMSF) for 10 min on ice. Nuclei were pelleted by centrifugation at 2000g for 5 min, resuspended in nuclear lysis buffer (50 mM Tris pH 8.1, 10 mM EDTA, 1% SDS) containing the same protease inhibitors as in the cell lysis buffer, and incubate on ice for 10 minutes. Chromatin extracted following nuclear lysis was sonicated using a Bandelin Sonopuls ultrasonic homogenizer to an average fragment size of 150 to 500 bp, as determined by agarose gel electrophoresis. The samples were diluted into five volumes of ChIP dilution buffer (0.01% SDS, 1.1% Triton X-100, 1.2 mM EDTA, 16.7 mM Tris pH 8.1, 167 mM NaCl, plus protease inhibitors) and incubated with 100 μl of a salmon sperm DNA/protein A-sepharose slurry for 1 h at 4°C, with mixing. Ten percent of chromatin was withdrawn (Input) and processed as the immunoprecipitated chromatin. Aliquots of chromatin containing 25 μg of DNA were incubated overnight at 4°C in the absence of antibodies or either with the anti-acetyl histone H3K9 or the anti-HDAC1 antisera purchased from Upstate Cell Signaling Solutions (cat# 07–352 and cat# 06–720, respectively). The immune complexes were adsorbed to protein A-sepharose beads, which were then sequentially washed with a low salt wash buffer (0.1% SDS, 1% Triton X-100, 2 mM EDTA, 20 mM Tris pH 8.1, 150 mM NaCl), a high salt wash buffer (0.1% SDS, 1% Triton X-100, 2 mM EDTA, 20 mM Tris pH 8.1, 500 mM NaCl), a LiCl wash buffer (0.25 M LiCl, 1% NP40, 1% deoxycholate, 1 mM EDTA, 10 mM Tris pH 8.0), and twice in 1x TE buffer. The immune complexes were then eluted with the elution buffer (1% SDS, 0.1 M NaHCO_3_), digested with RNase at 37°C, and treated with proteinase K in 0.3 M NaCl at 65°C for 4 h to reverse the cross-links. DNA from chromatin samples was extracted with phenol/chloroform, precipitated with ethanol and dissolved in 50 μl of water. Finally, DNA samples were quantified by readings in a Qubit Fluorometer (Invitrogen) using the Quant-iT dsDNA HS assay kit (Invitrogen).

The enrichment of either *hbox12* or *nodal* gene promoter sequences in 100 pg aliquots of ChIPed DNA and input controls was examined by qPCR, as described above, using the oligonucleotide primers indicated in the S1 Table. Ct values obtained for each IP sample were normalized to Ct values of the Input, which represented one hundredth of the total chromatin in the IP samples before the precipitation with a specific antibody. Then, the percent of Input values were calculated separately for each of the three replicates of an IP sample using the following formula 2x100^normalized Ct^, and finally averaged.

### Microinjection, embryo manipulation and imaging

Microinjection was conducted as described [68–70]. Approximately 5000 molecules of the linearized *phbox12*-GFP transgene were injected per zygote. The *phbox12*-GFP transgene corresponds to the construct originally referred to as 1.45GFP [49].

Capped HD mRNA was synthesized from the linearized pCS2 construct using the mMessage mMachine kit (Ambion). Approximately 1–2 pl of the purified RNA were then injected at 0.1–0.2 pg/pl. For all experiments, several hundreds of injected embryos were observed and each experiment was repeated at least three times with different batches of eggs.

Injected embryos at the desired stage were harvested, mounted on glass slides and examined under a Leica DM-4500B upright fluorescent microscope. Digital images were captured and processed using Adobe Photoshop CS6.

### Embryo disaggregation and flow cytometry

Following injection of *hd-gfp* mRNA into a single blastomere at the 4-cell stage, embryos were reared in the presence of TSA 50 nM and harvested at the early blastula stage. Approximately 300 embryos were transferred into a single well of a 96-well round-bottom plate and disaggregated by incubation in 0.22 μm filtered Ca^2+^-free sea water containing 1% BSA, followed by repeated forced mixing using a P200 micropipette (Eppendorf). The individual cell suspension was collected and immediately sorted using a FACSAria III Flow Cytometer (BD Biosciences) set to 4°C. A non-fluorescent cell population derived from undisturbed embryos at the early blastula stage was run on the cytometer as a negative control. Total RNA was extracted separately from both the sorted fluorescent and non-fluorescent fractions, as well as from whole uninjected TSA-treated embryos, and fold difference in *nodal* mRNA abundance assessed by qRT-PCR with respect to cDNA samples derived from control undisturbed embryos.

## Supporting Information

S1 Table List of gene specific oligonucleotides used in the qRT-PCR. (DOC)
