# Supplementary material for: Ectopic hbox12 Expression Evoked by Histone Deacetylase Inhibition Disrupts Axial Specification of the Sea Urchin Embryo
Source: PLoS One. 2015 Nov 30;10(11):e0143860. doi: 10.1371/journal.pone.0143860 (PMC4664418; doi:10.1371/journal.pone.0143860)
Supplement: S1 Table — (DOC) [file pone.0143860.s001.doc]

**S1 Table. List of gene-specific oligonucleotides used in the qPCR analyses.**

| Target gene | Forward (F) and Reverse (R) sequences (5’ to 3’) | Length | Amplicon size (bp) | Figures in which oligonucleotide was used |
| --- | --- | --- | --- | --- |
|  |  |  |  |  |
| *hbox12 (coding)* | F: ACGTCTTCGTCGAGCATCTC  R: GCATGGTGCCTTTCGCTTACG | 20  21 | 124 | 1C |
| *hbox12 (promoter)* | F: GGAGAGAAGTTGTGAGAGAGC  R: AGGCCTATTATGATTAATCTCAT | 21  23 | 110 | 2B and 2C |
| *nodal (coding)* | F: ACAACCCAAGCAACCACGCA  R: CGCACTCCTGTACGATCATG | 20  20 | 174 | 4B and 4G |
| *nodal (promoter)* | F: CGAGAACCGTTGCTTCTATTC  R: CAGACACTACCTGCCCCTTA | 21  20 | 146 | 3C |
| *gfp* | F: AGGGCTATGTGCAGGAGAGA  R: CTTGTGGCCGAGAATGTTTC | 20  20 | 152 | 2E |
| *mbf-1*^§^ | F: ATGACACAGCCTGGAGCT  R: TACCAAGGAAGTGGGTGT | 18  18 | 102 | 1C, 2E, 4B, and 4G |
| *cit-ox*^§^ | F: GTTGGGGTTAATCTAACATTCTTC  R: GAGGGTATAGGCATCTGGATAG | 24  22 | 93 | 1C, 2E, 4B, and 4G |
|  |  |  |  |  |

^§^ The *H2A* histone modulator binding factor (*mbf-1*), *z12*, or a *cytochrome-oxidase* (*cyt-ox*) mRNA were used to normalize all data.
